# Investigating antidiabetic drug targets as potential therapeutic modulators for schizophrenia

**DOI:** 10.1007/s00213-025-06951-3

**Published:** 2025-11-10

**Authors:** Yu-Sheng Huang, Xu Lin, Ya-Juan Xu, Gui-Bing Chen

**Affiliations:** 1 Xiamen Xianyue Hospital, Xianyue Hospital Affiliated with Xiamen Medical College, Fujian Psychiatric Center, Fujian Clinical Research Center for Mental Disorders, No.387~399 of Xianyue Road, Xiamen, Fujian 361000 China; 2 The School of Clinical Medicine, Fujian Medical University, Fuzhou, Fujian China

**Keywords:** Schizophrenia, Drug targets, Antidiabetic drugs, Gene expression, Mendelian randomization

## Abstract

**Rationale:**

Certain types of antidiabetic drugs (ADs) have been proven to improve cognitive functions and symptom dimensions in schizophrenia (SCZ).

**Objectives:**

Schizophrenia is a major contributor to social functional impairment, and antipsychotics (APs) remain the cornerstone of its treatment. Investigating the potential of antidiabetic drugs as APs could significantly advance therapeutic strategies for schizophrenia.

**Methods:**

To assess this potential, we employed an integrated analytical framework that included two-sample Mendelian randomization (MR) using genetic proxies for antidiabetic drug targets, multivariable MR (MVMR), and colocalization analyses. Furthermore, gene and drug enrichment analyses alongside molecular docking studies were performed to elucidate underlying mechanisms and identify candidate drugs. The analysis utilized summary statistics from genome-wide association meta-analyses (GWAS) of schizophrenia, as well as gene expression data from the eQTLGen consortium.

**Results:**

A total of fourteen drug targets were identified among eight major antidiabetic drugs. Notably, RXRB, a target of thiazolidinediones (TZDs), exhibited a strong inverse association with schizophrenia risk (OR: 0.28, 95% CI: 0.21–0.38, *P* < 0.001). Other significant targets included GPD1 (targeted by metformin), LRP2 (insulin), SLC5A2 (SGLT2 inhibitors), and ABCC8/KCNJ11 and INS (sulfonylureas). Primary and secondary MR analyses confirmed the associations of RXRB, INS, LRP2, and SLC5A2 with gene expression levels. Drug enrichment and molecular docking analyses identified bezafibrate as a promising candidate for repurposing as an AP.

**Conclusion:**

Antidiabetic drugs may possess therapeutic potential in schizophrenia by modulating specific drug-related pathways.These findings provide new theoretical support for the selection of ADs in patients with schizophrenia comorbid with diabetes, and also offer new insights for the future development of APs.

**Supplementary Information:**

The online version contains supplementary material available at https://doi.org/10.1007/s00213-025-06951-3.

## Introduction

Schizophrenia is a chronic mental illness with a prevalence of approximately 1% (Borelli and Solari 2019). It is associated with brain aging, including cortical atrophy (Takayanagi et al. 2020), dendritic spine loss (Glausier and Lewis 2013; Moyer et al. 2015), and cognitive impairment (Jeste et al. 2011). Life expectancy in schizophrenia is over 10 years shorter than the general population due to increased suicide, accidental deaths, and higher risks of cardiovascular and respiratory diseases (Correll et al. 2022; Saha et al. 2007). Additionally, schizophrenia patients exhibit higher rates of smoking (Brown et al. 1999), diabetes (Pillinger et al. 2017), obesity (Allison et al. 1999), and hyperlipidemia (Henderson et al. 2015), making effective treatments critical.

Antipsychotic treatments are closely linked to metabolic syndrome, with a prevalence of 35.77% among patients on stable medication (Ma et al. 2024). Antipsychotics induce insulin resistance, glucose depletion, and mitochondrial dysfunction, promoting metabolic syndrome (Manta et al. 2025),. Even before antipsychotic treatment, impaired glucose tolerance is observed in first-episode schizophrenia patients, with a higher incidence of type 2 diabetes (T2DM) compared to treated patients (Li et al. 2024; Fang et al. 2024). These findings have spurred interest in repurposing antidiabetic drugs as potential antipsychotic agents (Alagiakrishnan and Halverson 2024). Metformin has been shown to improve cognitive and symptomatic dimensions of schizophrenia, and drugs targeting insulin pathways may affect disease progression (Battini et al. 2023; Shao et al. 2023; Devine et al. 2024). A novel sulfonylurea compound has also demonstrated promise as an antipsychotic treatment (Zhang et al. 2022).

To investigate the causal effects of antidiabetic drugs on schizophrenia, Mendelian Randomization (MR) provides a robust approach using genetic variants (SNPs) as instrumental variables (Sanderson et al. 2022). This study employs MR to explore the potential of antidiabetic drugs in modifying schizophrenia progression by targeting drug-related biological pathways. By integrating metabolic and neuropsychiatric research, this study reveals the potential of antidiabetic drugs to act as antipsychotic agents, provides new guidance for treatment medication selection in patients with schizophrenia comorbid with diabetes.

## Research methods and materials

### Data sources

#### Identification and validation of antidiabetic drug targets

**Fig. 1:**
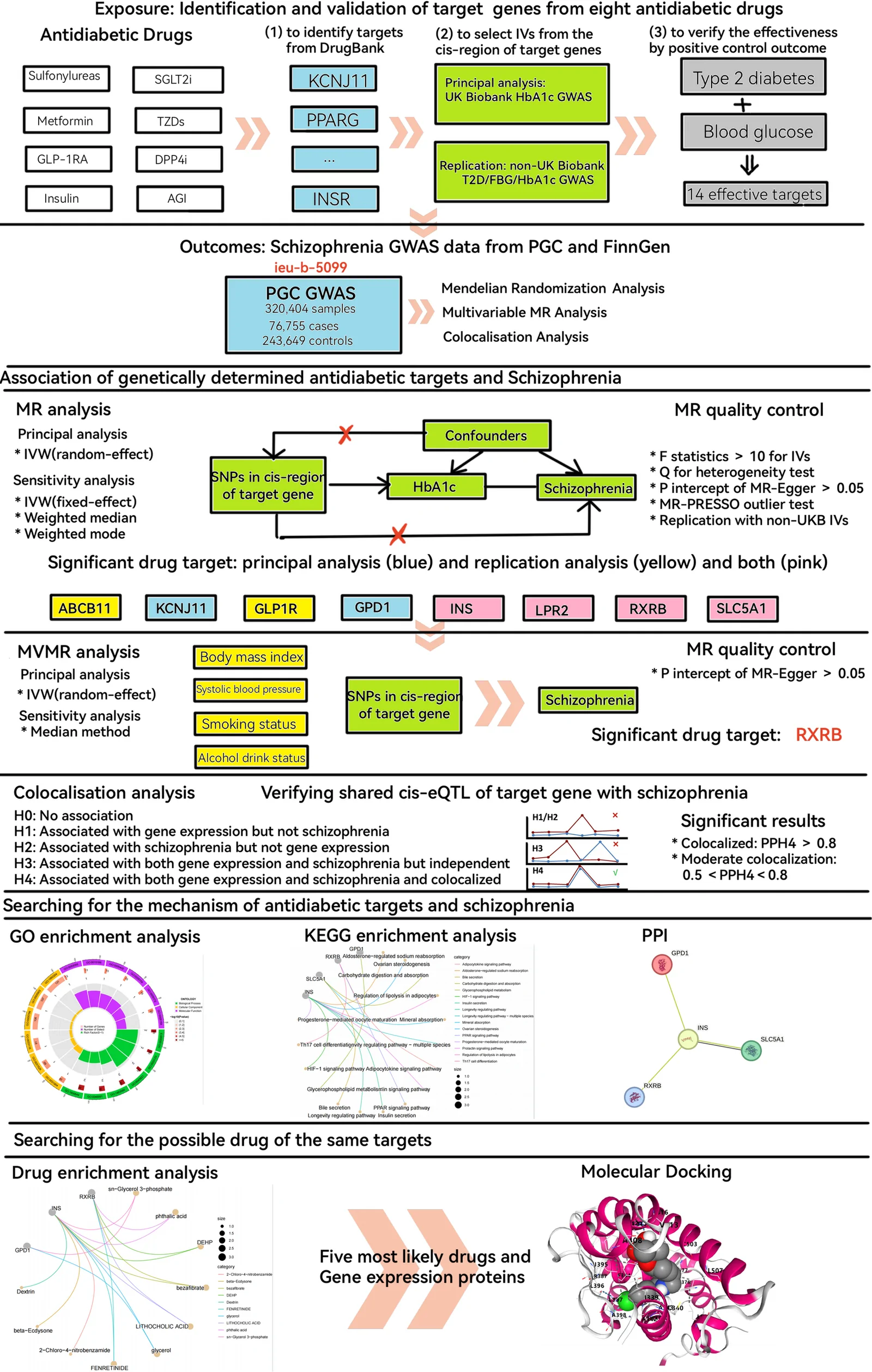
Flowchart illustrating the systematic approach for identifying the association between antidiabetic drug targets and schizophrenia. This flowchart describes the Mendelian randomisation framework used to assess the causal effects of antidiabetic drug targets on schizophrenia. If two adjacent target genes shared the same IVs due to overlapping cis-regions, these genes were combined and marked with a slash, such as “ABCC8/KCNJ11”. Abbreviations: MR, Mendelian randomisation; MVMR, multivariable Mendelian randomisation; IVs, instrumental variables; GWAS, genome-wide association studies; IVW, inverse variance weighted; T2DM, type 2 diabetes mellitus; HbA1c, haemoglobin A1C; SNPs, single nucleotide polymorphisms; eQTLs, expression quantitative trait loci; H0–H4, Hypotheses 0–4; GO, Gene Ontology; KEGG, Kyoto Encyclopedia of Genes and Genomes; PPI, Protein-protein interaction

The procedural workflow of the study is illustrated in Fig. 1. The identification of antidiabetic drug targets was performed in three distinct steps. First, gene targets for eight approved clinical antidiabetic drugs were identified using the DrugBank pharmacogenomics database (https://go.drugbank.com/) (Supplemental Tables, Table S1). The details of these gene targets are listed in Table S2. Second, instrumental variables (IVs) for these targets were derived from a genome-wide association study (GWAS) on hemoglobin A1c (HbA1c) levels conducted in the UK Biobank population (Mbatchou et al. 2021), with selection criteria of *P* < 5 × 10^−8 and r^2 < 0.2 (Yarmolinsky et al. 2023). This analysis identified IVs for seven primary antidiabetic drug targets, including sulfonylureas, metformin, alpha-glucosidase inhibitors (AGIs), thiazolidinediones (TZDs), GLP-1 receptor agonists (GLP-1 RAs), insulin and its analogs, and sodium-glucose cotransporter-2 inhibitors (SGLT2is). Although dipeptidyl peptidase-4 inhibitors (DPP4is) are also associated with antidiabetic drugs, they were excluded due to the lack of IVs meeting the specified criteria. For cases where overlapping cis-regions resulted in identical IVs being shared by adjacent target genes, the genes were merged and represented with a slash (e.g., “ABCC8/KCNJ11”). Additionally, the IVs for ESRRA were excluded automatically by the harmonise_data function in the TwoSampleMR package because of palindromic strand issues.

IVs were further supplemented from other GWAS datasets beyond the UK Biobank. Since no single dataset included IVs for all HbA1c-, glucose-, or type 2 diabetes mellitus (T2DM)-related drug targets, six alternative datasets were used. These comprised HbA1c GWAS data from MAGIC (Soranzo et al. 2010) and the Within Family GWAS consortium (Howe et al. 2022), T2DM GWAS data from DIAGRAM (DIAGRAM et al., 2014) and FinnGen, and fasting glucose GWAS data from MAGIC (Scott et al. 2012). All these datasets primarily included individuals of European ancestry and were obtained from the EU International Partnership Forum platform (Table S3).

Finally, the identified drug targets were validated against four GWAS datasets related to glucose levels and T2DM as positive controls (Table S3). KCNJ1 was excluded from subsequent analyses because it was not significantly associated with any positive control outcomes (Table S4). A comprehensive summary of IVs and their corresponding effects for each drug target is provided in Table S5.

#### Determination of study outcomes

The genetic association of schizophrenia was obtained from the Psychiatric Genomics Consortium (PGC). It primarily included samples of European ancestry, comprising 320,404 samples (76,755 cases and 243,649 controls). Routine regression analysis accounted for covariates such as age, gender, genotyping array, and the first twenty principal components. Schizophrenia was diagnosed based on psychiatric evaluation and medical records, according to the Diagnostic and Statistical Manual of Mental Disorders-IV (DSM-IV) or ICD-10 (Lam et al. 2019). We used the European ancestry sample cohort for subsequent analysis.

### Research method

#### Two-sample MR and replication analysis

We employed MR to estimate the causal effects of each antidiabetic drug target on the phenotype of schizophrenia. To distinguish the effects of blood glucose changes from the effects of antidiabetic drugs on schizophrenia risk, we used MR to focus on the genetic proxies of drug targets rather than directly measuring blood glucose levels. This approach helps isolate the specific effects of drug targets from blood glucose levels. According to the causal diagram for MR shown in Fig. 1, the method must satisfy three assumptions: (1) IVs should be associated with the exposure; (2) IVs should influence the outcome only through the exposure (lower red cross in Fig. 1); and (3) IVs should not be associated with any confounders (upper red cross in Fig. 1). To meet these assumptions, we employed several methods. The primary analysis method was the inverse-variance weighted (IVW) MR with random effects (Burgess et al. 2013). To ensure the robustness of our findings, we also conducted sensitivity analyses using fixed-effect IVW, weighted median, and weighted mode methods. These methods were used to address potential violations of the IV assumptions. We compared the odds ratios (ORs) and p-values obtained from all four methods to assess the stability and validity of the findings. Random-effects IVW was used to mitigate potential biases due to high heterogeneity among multiple IVs. Heterogeneity among IVs was quantified using the Q test. The weighted median method provided robust estimates even in the presence of some invalid genetic instruments (if the proportion of invalid instruments was less than 50%) (Bowden et al. 2016a, b). The weighted mode estimator was considered reliable when most similar causal effect estimates were derived from valid IVs (Hartwig et al. 2017). Results that were significant across these methods were considered robust associations. Additionally, we used the intercept from MR-Egger regression to test for horizontal pleiotropy (Burgess and Thompson 2017). All MR analyses were conducted using R version 4.4.1 (https://www.r-project.org/) with the “TwoSampleMR” package. We also conducted MR-PRESSO analysis to test and correct for the presence of specific IV outliers (potentially pleiotropic SNPs) as another sensitivity analysis (Verbanck et al. 2018).

To address potential sample overlap between the schizophrenia GWAS data and the primary HbA1c GWAS used in our two-sample MR analysis, we conducted replication analyses to further validate the robustness of our findings. For these analyses, we selected IVs (in the cis-region (± 500 kb) of the targets using the same parameters *P* < 5 × 10^−8 and r^2 < 0.2) for target genes/drugs from non-UK Biobank exposure GWAS datasets to ensure there was no potential sample overlap.

#### Multivariable MR (MVMR) analysis

Furthermore, to evaluate the robustness of the MR results, we performed multivariable MR to analyze whether confounding factors might influence the findings (Burgess and Thompson 2015). This analysis also used IVs from the cis-region (cis-window: ±500 kb) of each drug target and adjusted for body mass index, systolic blood pressure, smoking status, and alcohol consumption status, using the same parameters (*P* < 5 × 10^−8, r^2 < 0.2). The strength of the IVs was determined using the F-statistic, calculated as $\:{\mathrm{F}}_{\mathrm{j}}\:=\:{{\upgamma\:}}_{\mathrm{j}}^{2}/{{\upsigma\:}}^{2}{\mathrm{X}}_{\mathrm{j}}$, where $\:{{\upgamma\:}}_{\mathrm{j}}$ is the estimate of the SNP-exposure association, and $\:{{\upsigma\:}}^{2}{\mathrm{X}}_{\mathrm{j}}$ is the standard error of this association (Bowden et al. 2016a, b). In our study, all observed F-statistics comfortably exceeded the threshold of F > 10, indicating no weak instrument bias. We used the MR-Egger intercept test to evaluate pleiotropic effects and the Q test to assess heterogeneity in the IVW method. When multiple target genes’ IVs were available for a single antidiabetic drug, combined IVs were used in the MR analysis to demonstrate the overall effect (detailed in Table S6).To further isolate the independent effects of each gene while controlling for potential confounding factors, we queried the GWAS Catalog for disease associations at each gene locus and supplemented the results in Table S15. The GWAS Catalog is available at: https://www.ebi.ac.uk/gwas/.

#### Colocalisation analysis

Colocalisation analysis was performed to determine whether the association between specific gene expression and schizophrenia could be attributed to the same causal genetic variants. This analysis is based on a Bayesian model with posterior probabilities for five hypotheses (PPH): H0 indicates no association with either trait; H1 indicates association with only the first trait; H2 indicates association with only the second trait; H3 indicates different causal variants associated with each trait; and H4 indicates the same causal variant associated with both traits (Foley et al. 2021). The coloc.abf algorithm in the R package (http://cran.r-project.org/web/packages/coloc) was used (parameter setting: p1 = 1 × 10^−4, p2 = 1 × 10^−4, p12 = 1 × 10^−3). We considered PPH (H4) greater than 0.8 as strong evidence for colocalisation between gene expression targets and schizophrenia, while PPH (H4) greater than 0.5 indicated moderate colocalisation (Fig. 1).

#### Gene ontology (GO) enrichment analysis

To gain a comprehensive understanding of the biological roles of significant drug target genes and regulatory proteins identified via MR analysis, we utilized the g: Profiler database to perform Gene Ontology (GO) and pathway enrichment analyses (https://reactome.org/). GO analysis characterizes gene functions across three categories: cellular components (CC), molecular functions (MF), and biological processes (BP). Functional categories with P values < 0.05 were considered significantly enriched (Gene Ontology Consortium, 2015).

#### Kyoto encyclopedia of genes and genomes (KEGG) enrichment analysis

KEGG is a biological database that stores information on genes and genomes, metabolic pathways, and drugs. KEGG enrichment analysis is a bioinformatics method used to analyze the biological functions of a set of genes or proteins, primarily to explore the enrichment of a set of genes in different biological pathways. In KEGG enrichment analysis, we typically map the gene set of interest (e.g., differentially expressed genes, mutated genes) to KEGG pathways to determine whether these genes are significantly enriched in specific pathways. By using the “clusterProfiler” R package, we mapped the significant drug target gene set to known pathways in the KEGG database, calculating whether these genes are significantly more prevalent in certain pathways than would be expected by chance. The enrichment degree of the pathways is calculated through statistical methods such as hypergeometric distribution or Fisher’s exact test, thereby identifying biological pathways associated with the gene set.(Kanehisa et al. 2016).Furthermore, we utilized 54 GTEx (Genotype-Tissue Expression Project) tissues to perform genome-wide tissue-specific enrichment analysis to validate the tissue specificity and brain-related specificity of these gene expression patterns(Table S16).

#### Protein-protein interaction (PPI) network analysis

Protein-protein interaction (PPI) network analysis serves as a powerful approach to exploring the interactions between proteins within cellular systems. Using STRING and Cytoscape software, we mapped interactions among the proteins encoded by the significant drug target genes. A network was constructed based on a confidence interaction score threshold of 0.15, highlighting the key biological processes and signaling pathways. Within this network, each node represents a protein, while edges (connections) depict the interactions between these proteins. PPI network analysis enables the identification of critical proteins, functional modules, and protein clusters involved in specific biological processes (Szklarczyk et al. 2017).

#### Drug enrichment analysis and molecular Docking

Drug enrichment analysis is a method for studying the association between a specific set of genes or proteins and known drugs from a systems biology perspective. Through drug enrichment analysis, we can identify drugs that may be related to specific biological processes or diseases, providing clues for drug screening and repurposing. Drug enrichment analysis is primarily based on gene expression profiles, target similarity, or known drug-gene interaction data. Molecular docking is a computational simulation method used to predict the binding mode and binding strength between small molecules (e.g., drug molecules) and biological macromolecules such as proteins. Molecular docking can help researchers assess the binding affinity, sites, and interaction modes between drug molecules and target proteins.

The principle of molecular docking is to find the conformation with the lowest binding energy by calculating the geometric shape and energy (e.g., hydrogen bonds, hydrophobic interactions) of the interactions between compounds and proteins. Generally, the higher the docking score (lower binding energy), the more likely the compound is to effectively bind to the target protein.

We extracted drug and drug target information from DrugBank, used the “enrichplot” R package for drug enrichment analysis of significant drug target genes, and selected the five drugs with the smallest P-values for molecular docking with near-target gene proteins. Gene protein structures were obtained from the PDB database (Protein Data Bank) (https://www.rcsb.org/), small molecule drug structures were obtained from the PubChem database (https://pubchem.ncbi.nlm.nih.gov/), and online molecular docking was performed on the CB-DOCK2 website (https://cadd.labshare.cn/cb-dock2/index.php) (Xue et al., 2024).

## Results

### Effective antidiabetic drug targets using blood glucose and T2DM as positive control outcomes

Fourteen viable drug targets were identified that could harmonise with schizophrenia outcomes across seven antidiabetic drug groups: sulfonylureas (ABCB11, ABCC8/KCNJ11, CPT1A, INS, KCNJ1, KCNJ8/ABCC9, LRP2/ABCB11, and VEGFA/SLC29A1), TZDs (PPARG, RXRB, and VEGFA/SLC29A1), GLP1-RA (GLP1R), insulin and its analogues (LRP2/ABCB11), AGIs (GANC), metformin (GPD1), and SGLT2i (SLC5A1 and SLC5A2) (Table S4). The SNPs and corresponding effects for each drug target are shown in Table S5. This analysis revealed 14 targets significantly associated with blood glucose levels and T2DM, except for KCNJ1 (Table S4). The data processing and summary in this section refer to an article that explores antidiabetic drug targets as potential therapeutic targets for osteoarthritis.(Fu et al. 2024).

### Effects of genetically determined antidiabetic drug targets on schizophrenia through MR and MVMR

**Fig. 2:**
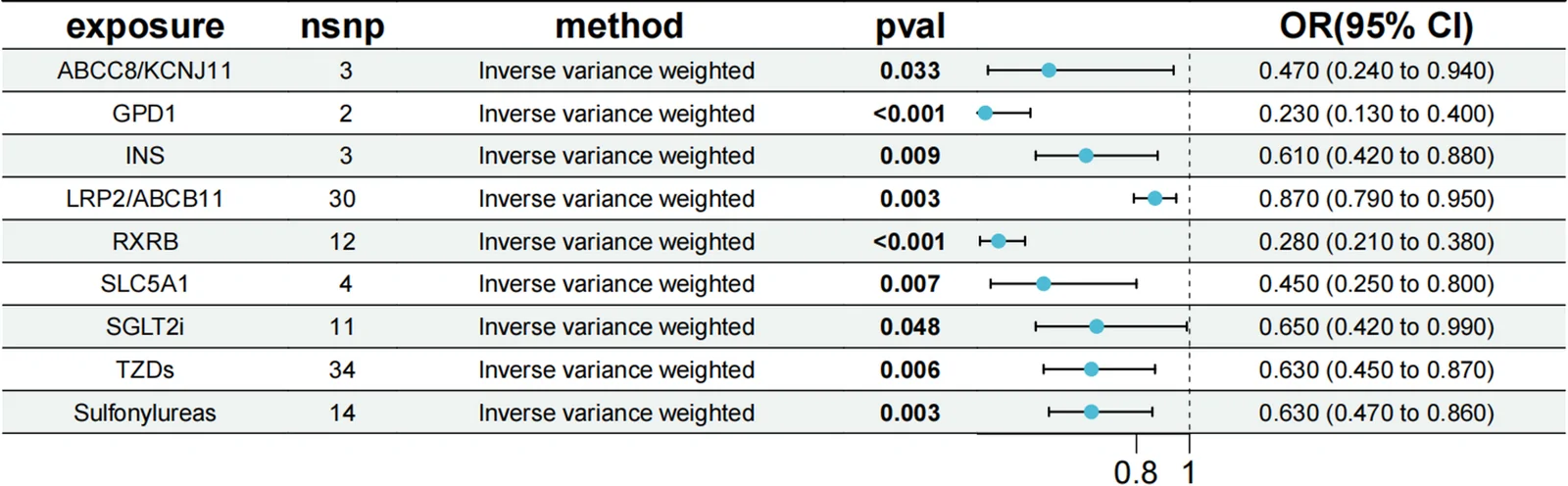
Results of the primary MR analysis

Under the primary analysis of IVW(random-effects), sensitivity analyses (fixed-effects IVW, weighted median, and weighted mode methods), and quality control measures (F-statistic for instrumental variables [IVs] > 10; Q test for heterogeneity < 50%; MR-Egger intercept P-value > 0.05), six significant drug targets were identified to be associated with schizophrenia in the MR analyses (Fig. 2).

In the primary analysis using random-effect IVW(Fig. 2), the sulfonylurea drugs targeting ABCC8/KCNJ11 and INS showed a significant association between the reduction in HbA1c levels and a decreased risk of schizophrenia. (OR: 0.47, 95% CI: 0.24–0.94, *P* = 0.033 for ABCC8/KCNJ11; OR: 0.61, 95% CI: 0.42–0.88, *P* = 0.009 for INS). The insulin target LRP2, which shares IVs with the sulfonylurea target ABCB11, also exhibited a consistent effect similar to the sulfonylurea target ABCC8/KCNJ11 (OR: 0.87, 95% CI: 0.79–0.95, *P* = 0.003 for LRP2). The TZDs targeting RXRB, metformin targeting GPD1, and SGLT2i targeting SLC5A1 were also significantly associated with a reduced risk of schizophrenia (OR: 0.28, 95% CI: 0.21–0.38, *P* < 0.001 for RXRB; OR: 0.47, 95% CI: 0.24–0.94, *P* = 0.033 for GPD1; OR: 0.45, 95% CI: 0.25–0.80, *P* = 0.007 for SLC5A1). Three drugs with multiple available target genes, including sulfonylureas, TZDs, and SGLT2i, were significantly associated with schizophrenia. The combined targets of these three drugs demonstrated a protective effect against schizophrenia.

The results obtained from different MR methods in sensitivity analyses were generally consistent with the primary findings, as shown in Table S6 and Figures S1–S4. Additionally, in the MR analysis, only a few target-outcome pairs detected outlier SNPs through the MR-PRESSO global test; however, the outlier-corrected results also supported the above findings, indicating limited bias from horizontal pleiotropy (Table S6).

In the replication analysis using non-UK Biobank IVs, the results for targets INS, RXRB, LRP2/ABCB11, and SLC5A1 were further replicated, avoiding potential sample overlap bias (Tables S7, Figures S5 and S6).

**Fig. 3:**
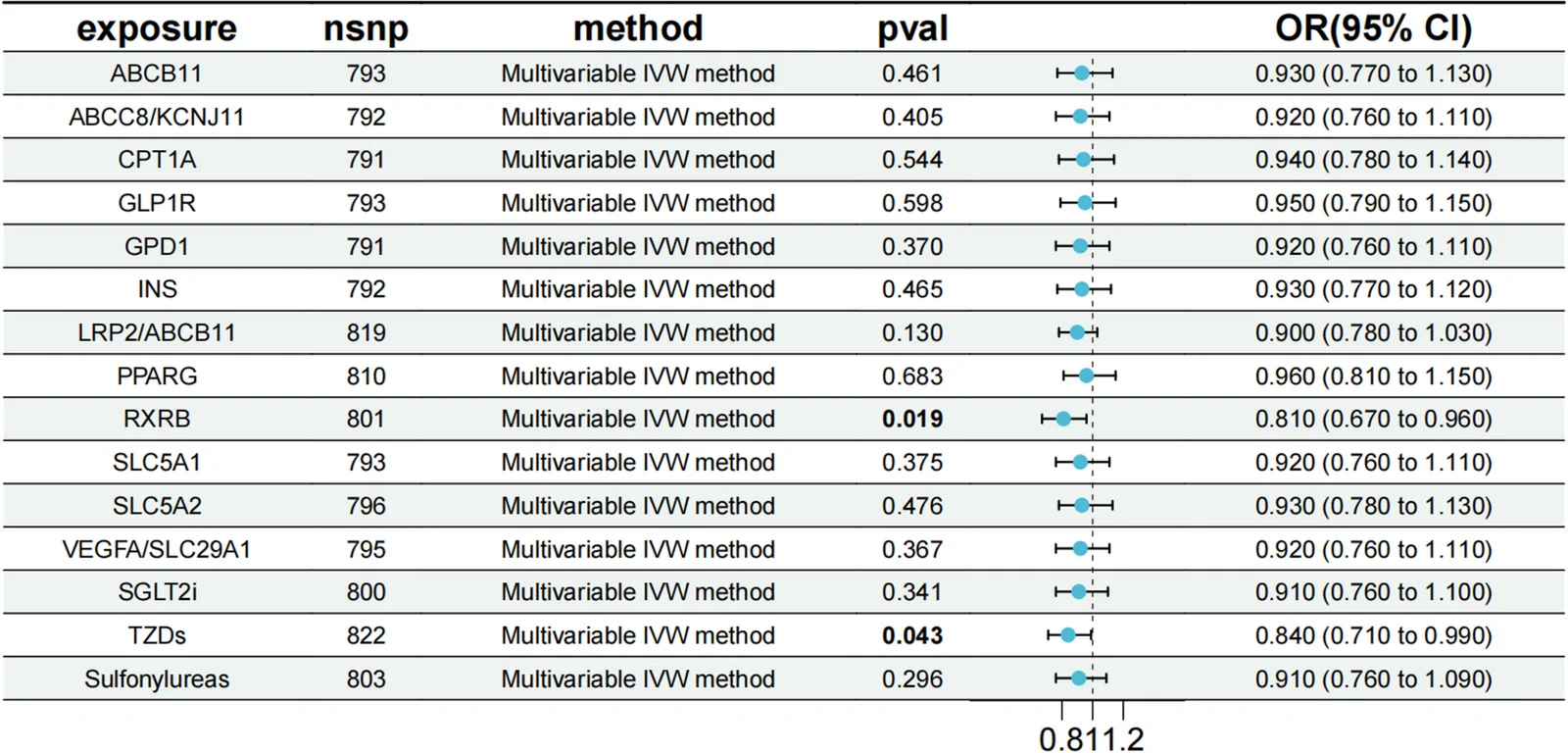
Results of the MVMR analysis

In the MVMR analysis, the impact of representative targets was further confirmed only for RXRB and its corresponding TZDs drugs (Fig. 3, Table S8, Fig. S7).

Additionally, the GWAS Catalog search results (Table S15) revealed that the ABCC8 gene influences schizophrenia through synaptic biology mechanisms (Trubetskoy et al. 2022). The INS gene contributes to schizophrenia by leveraging genetic overlap with brain morphology (van der Meer et al. 2022). No disease-associated loci were identified for the SLC5A2 gene.

### Colocalisation of the putative proteins with schizophrenia

The colocalisation analysis presented results of these genes with schizophrenia (Table S9). We considered PPH (H4) greater than 0.8 as strong evidence for colocalisation between gene expression targets and schizophrenia, while PPH (H4) greater than 0.5 indicated moderate colocalisation。However, regrettably, no evidence of colocalization was observed between drug-target genes and schizophrenia based on the aforementioned criteria.

### GO enrichment, KEGG pathway enrichment, drug enrichment and PPI network analysis, GTEx tissue distribution analysis

**Fig. 4:**
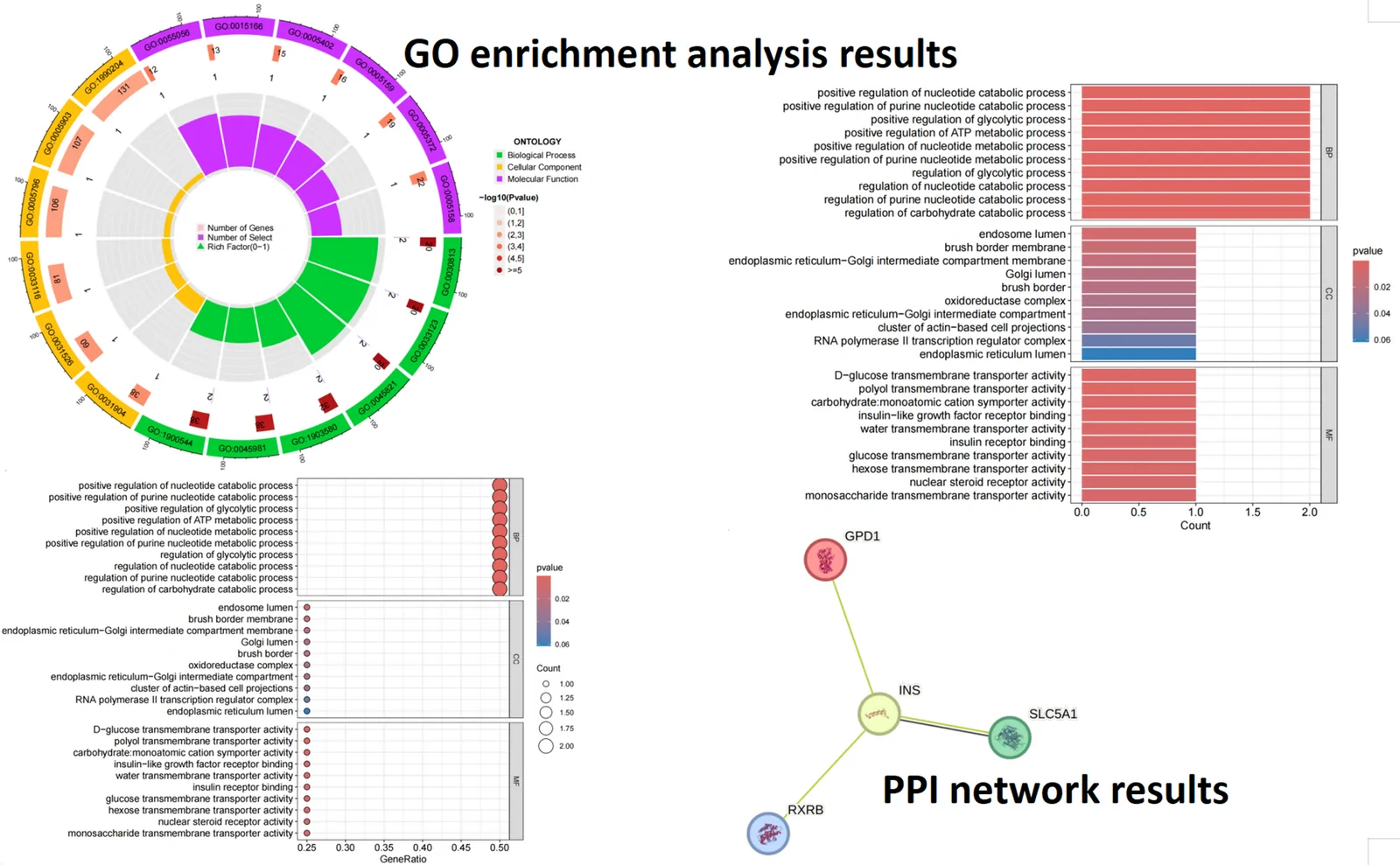
GO enrichment analysis results and PPI network results

GO enrichment analysis revealed that antidiabetic drug targets were primarily enriched in processes related to nucleotide and carbohydrate catabolism, glucose transmembrane transporter activity, and insulin-like growth factor receptor binding(Fig. 4). Proteins were localized to the endosome lumen and brush border membrane (Fig. 4, Table S10).

**Fig. 5:**
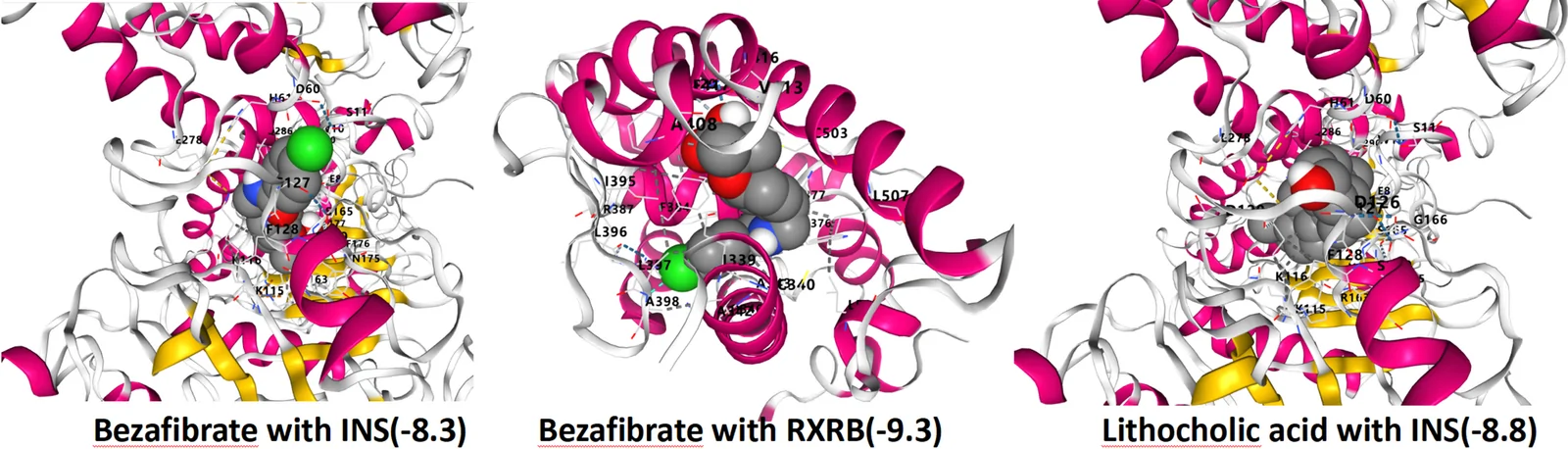
Molecular docking results of significant drug enrichment analysis

KEGG analysis identified five key pathways: “Aldosterone-regulated sodium reabsorption,” “Ovarian steroidogenesis,” “Carbohydrate digestion and absorption,” “Regulation of lipolysis in adipocytes,” and “Mineral absorption” (Fig. S8, Table S11).

The GTEx tissue distribution analysis (Table S16) revealed significant enrichment of the studied drug-target genes in gastric and pancreatic tissues.

The six antidiabetic drug targets were submitted to the STRING database for PPI analysis. A PPI network consisting of four nodes and three edges was constructed using Cytoscape 3.10.1. In the network, nodes represent target proteins, and lines between nodes represent protein-protein interactions. The size of the nodes corresponds to the degree of protein targeting in the network. The core target identified was INS (Fig. 4, Table S12).

The drug enrichment analysis revealed the enrichment results of the six significant drug gene targets from the MR results in other drugs (Fig. S9, Table S13). The five drugs with the highest degree of enrichment were “sn-Glycerol 3-phosphate,” “Phthalic acid,” “DEHP,” “Bezafibrate,” and “Lithocholic acid,” which will undergo subsequent molecular docking.

### Molecular docking results

Molecular docking results demonstrated that two drugs showed binding energy less than − 8 kcal/mol with their respective gene-expressed proteins: bezafibrate with INS (−8.3), bezafibrate with RXRB (−9.3), and lithocholic acid with INS (−8.8). The molecular docking structures are shown in Fig. 5, while the docking results for the remaining drugs are detailed in Table S14.

## Discussion

Predicting novel therapeutic targets for mental disorders using methods such as drug-target Mendelian randomization (MR), colocalization analysis, molecular docking, and enrichment analysis has become a research hotspot in recent years. Some researchers, employing approaches similar to this study, have identified potential therapeutic targets for schizophrenia, including IGLON5, PTK7, LIMA1, and HBEGF. Through molecular docking, drugs exhibiting high binding affinity to these targets were identified, such as medroxyprogesterone acetate for HBEGF (Xiong et al. 2025). Another study similarly integrated genetic methods (Mendelian randomization, colocalization analysis, Summary-based Mendelian Randomization - SMR) and bioinformatic tools (drug prediction, molecular docking, single-cell analysis) to screen and validate potential drug targets for schizophrenia (SCZ), specifically highlighting FGFR1 as the most promising candidate target (Lian et al. 2025). Notably, a contemporaneous study by Yuan et al. also investigated antidiabetic drug targets for psychiatric disorders using MR but with key distinctions from our work(Yuan et al. 2025). While both studies employ genetic methods, Yuan et al. utilized brain-specific QTL data and focused on targets like GANC for bipolar disorder and ABCC8 for schizophrenia. In contrast, our study specifically targets schizophrenia using blood eQTL data, identifying distinct candidates such as RXRB, INS, LRP2, and SLC5A2, and further extends the analysis to drug repurposing via molecular docking. Although these findings await confirmation through animal or clinical studies, they provide valuable guidance for drug selection in schizophrenia patients with comorbid somatic conditions and serve as an initial screening step for the future development of novel antipsychotic drugs.

In this study, we employed Mendelian randomization (MR) analysis to investigate the causal relationship between antidiabetic drug targets and schizophrenia. Drug-target MR results indicated that a significant proportion of antidiabetic drug targets, including those for thiazolidinediones (TZDs), sulfonylureas, sodium-glucose cotransporter 2 inhibitors (SGLT2i), insulin, and metformin, exhibit antagonistic effects against schizophrenia. In multivariable Mendelian randomization (MVMR) analysis, after controlling for confounding factors such as BMI, blood pressure, smoking status, and alcohol use, the TZD-targeted RXRB gene (Retinoid X Receptor Beta) still demonstrated a significant protective effect, making it the primary focus of our investigation.

The RXRB gene, a key nuclear receptor within the retinoic acid (RA) signaling pathway, has been implicated in schizophrenia risk by genome-wide association studies (GWAS), which found enrichment of schizophrenia risk genes in RA pathway genes (including RXRB) (Wołoszynowska-Fraser et al. 2020). As a core member of the nuclear receptor superfamily, RXRB bidirectionally regulates metabolic and neuropsychiatric functions via lipid signaling. On the metabolic level, RXRB forms heterodimers with partners like PPARγ and LXR, directly regulating lipid homeostasis (e.g., activating cholesterol efflux genes ABCA1/APOC2), fatty acid oxidation, and glucose balance. Its dysregulation can lead to abnormal brain lipid content (e.g., increased RXRB accompanied by elevated cholesterol efflux in the prefrontal grey matter of schizophrenia patients) and overlaps genetically with metabolites like acylcarnitine and VLDL (Wnuk et al. 2019). On the neuropsychiatric level, RXRB-RAR complexes regulate neural development (e.g., prefrontal-thalamic connectivity and dendritic spine formation in primates), synaptic plasticity (hippocampal LTP/LTD and GluR1 receptor translation), and myelination through RA signaling gradients. Deficits in this pathway can impair working memory and lead to cognitive deficits (Shibata et al. 2021). Genetic evidence further links RXRB risk variants (e.g., rs12325245) and reduced expression to schizophrenia cognitive subtypes, decreased cerebellar volume, and abnormal grey matter covariance (Reay and Cairns 2020).Clozapine, frequently used for treatment-resistant psychosis despite its complex mechanism and severe side effects like agranulocytosis, remains clinically valuable due to its superior efficacy. Evidence suggests part of its mechanism may involve elevating brain and peripheral RA levels, thereby correcting the “RA signaling deficit” observed in schizophrenia patients and modulating synaptic plasticity. Beyond theoretical evidence, previous clinical trials also suggest RXRB-targeting improves schizophrenia symptoms. For instance, bexarotene, an RXR agonist originally used for cutaneous T-cell lymphoma, significantly improved positive symptoms in a randomized controlled trial, though it did not improve negative symptoms or quality of life (Lerner et al. 2016). This suggests retinoid receptor targeting (especially RXR) could be a novel strategy for schizophrenia, particularly for positive symptoms. Furthermore, prior research indicates TZDs can reduce dementia risk and slow progression in mild-to-moderate Alzheimer’s disease (AD) patients, especially those with comorbid metabolic disorders like diabetes (Pérez and Quintanilla 2015). This finding aligns with our conclusion of TZDs’ antagonism against schizophrenia, hinting at their potential protective effects on brain function.

Regarding other antidiabetic drugs—sulfonylureas, SGLT2i, insulin, and metformin—while their corresponding targets yielded negative results in our MVMR analysis, previous literature suggests possible relevance to schizophrenia treatment through various mechanisms. Although metformin was not validated in replication MR, numerous studies have shown its efficacy in improving cognitive and symptom dimensions in schizophrenia (Battini et al. 2023; Shao et al. 2023). One clinical study found sulfonylureas improved motor and cognitive function in neonates with KCNJ11 mutations, with 47% of patients reporting improvements in muscle tone, attention, and speech (Letourneau and Greeley 2019). Sulfonylureas may also attenuate neuroinflammation by inhibiting NLRP3 (as demonstrated by AMS-17) (Zhang et al. 2022), a mechanism highly relevant to schizophrenia pathogenesis (Buckley 2019). Another study indicates that central nervous system (CNS) insulin not only regulates metabolism (e.g., glucose homeostasis, feeding) but also influences cognition, dopaminergic signaling, and neuroinflammation, showing pathological links to schizophrenia (Agarwal et al. 2020). The Akt/GSK3β pathway represents a key intersection between insulin signaling and schizophrenia pathology, regulating glucose metabolism, dopamine signaling, and synaptic plasticity (Liu et al. 2013). Intranasal insulin has been proposed as a non-invasive strategy targeting brain insulin pathways, potentially offering new avenues for managing schizophrenia comorbidities (Agarwal et al. 2020). While literature directly supporting SGLT2i for improving schizophrenia symptoms is currently lacking, our drug-target MR study suggests a potential antagonistic effect, providing directional evidence for future research.

Pathway analysis revealed that antidiabetic drug target genes are enriched in processes related to nucleotide and carbohydrate metabolism, glucose transmembrane transport, and insulin-like growth factor receptor binding. Given that schizophrenia is associated with cerebral metabolic abnormalities, dysregulated insulin-like growth factor levels, and mitochondrial dysfunction, this suggests antidiabetic drugs may ameliorate symptoms by influencing these pathways. A peripheral-CNS interaction mechanism may exist, whereby these drugs indirectly modulate brain function and neurodevelopment via systemic metabolic pathways (e.g., insulin sensitivity, inflammatory responses), thereby affecting schizophrenia risk (Zhang et al. 2024; Xiong et al. 2024; Kopylov et al. 2023; Yang et al. 2024; Arinami et al. 2023; Papageorgiou and Filiou 2024). KEGG analysis identified pathways such as aldosterone-regulated sodium reabsorption and mineral absorption, previously linked to schizophrenia (Paba et al. 2011; Uddin et al. 2021). Protein-protein interaction analysis revealed an insulin (INS)-centered network, suggesting antidiabetic drugs may influence schizophrenia by modulating related metabolic processes. Drug enrichment and molecular docking analyses identified compounds like bezafibrate as candidate drugs for schizophrenia treatment, potentially mitigating antipsychotic-induced metabolic side effects and modulating brain transposable elements (Wei et al. 2019; Ferguson et al. 2018). While clinical studies on bezafibrate for schizophrenia are not yet found, animal models demonstrate its neuroprotective and mood-regulating effects (Lyu et al. 2022; Ribeiro et al. 2025; Seminotti et al. 2022; da Rosa-Junior et al. 2019; Wang et al. 2017).

All studies have limitations. We acknowledge that our research is predictive and requires further validation through animal and clinical trials. Despite rigorous measures, potential pleiotropy and bias in MR analyses cannot be entirely excluded. While sensitivity analyses (MVMR, MR-Egger regression, MR-PRESSO) were employed, future studies should integrate phenome-wide association studies (e.g., Steiger filtering) to explicitly assess associations between instrumental variables and non-glycemic phenotypes. Using HbA1c as a biomarker may introduce confounding from erythrocyte traits. Relying on cis-region SNPs for target genes limited our ability to fully adjust for multiple targets in MVMR. Although we successfully implemented MVMR using cis-region IVs, advancements in statistical methods are needed to address challenges in correcting for pleiotropy and overlapping drug targets (Holmes et al. 2021). Furthermore, limitations of publicly available summary-level data precluded direct assessment of the potential impact of sex differences, substance use, or SCZ + T2DM comorbidity on our results.

## Conclusions

Our study identifies a protective relationship between antidiabetic drugs and schizophrenia, suggesting that their effects extend beyond glucose regulation. These findings provide new theoretical support for the selection of ADs in patients with schizophrenia comorbid with diabetes, and also offer new insights for the future development of APs. Considering the heterogeneity of schizophrenia, future research should prioritize clinical trials to evaluate these drugs across diverse symptom profiles and investigate the molecular pathways mediating their effects to fully realize their potential as antipsychotic agents.

## Supplementary Information

Below is the link to the electronic supplementary material.

Supplementary Material 1 (PNG 123 KB)

Supplementary Material 2(PNG 127 KB)

Supplementary Material 3(PNG 252 KB)

Supplementary Material 4(PNG 288 KB)

Supplementary Material 5(PNG 118 KB)

Supplementary Material 6(PNG 313 KB)

Supplementary Material 7(PNG 285 KB)

Supplementary Material 8 (XLSX 1.14 MB)

Supplementary Material 9 (PNG 523 KB)

Supplementary Material 10 (PNG 469 KB)

Supplementary Material 10 (PNG 469 KB)

## Data Availability

All data generated or analyzed during this study are included in this published article.
